# Cell-penetrating peptide (CPP)-conjugated proteins is an efficient tool for manipulation of human mesenchymal stromal cells

**DOI:** 10.1038/srep04378

**Published:** 2014-03-14

**Authors:** Junghyun Jo, Soomin Hong, Won Yun Choi, Dong Ryul Lee

**Affiliations:** 1 Department of Biomedical Science, College of Life Science, CHA University, Seoul, Korea; 2 Fertility Center, CHA Gangnam Medical Center, College of Medicine, CHA University, Seoul, Korea; 3 Department of Biotechnology, Seoul Women's University, Seoul, Korea; 4 CHA Stem Cell Institute, CHA University, Seoul, Korea; 5 These authors contributed equally to this work.

## Abstract

Delivery of proteins has been regarded as the safest and most useful application in therapeutic application of stem cells, because proteins can regulate gene expression transiently without any genomic alteration. However, it is difficult to accurately measure efficiency or quantity of intracellular protein uptake. Here, we performed a comparison study of cell-penetrating peptide (CPP)-conjugated protein delivery system using seven arginine and Streptolysin O (SLO)-mediated system. To compare CPP- and SLO-mediated protein delivery systems, we used GFP and ESRRB protein, which is known to regulate pluripotency-related genes, for delivery into human bone marrow stromal cells (hBMSCs) and human testicular stromal cells (hTSCs). We found that CPP-conjugated protein delivery was more efficient, lower cytotoxicity, and higher biological activity than SLO-mediated protein delivery system. These results suggest that delivery of CPP-conjugated proteins is an efficient tool for introducing biologically active proteins into cells and may have important implications in clinical cell-based therapy.

Techniques that can alter the levels of gene expression and regulation by delivery of defined factors are useful tools in the understanding of cellular properties and biological processes. Many research groups have been working to improve intracellular delivery systems, and several techniques have been discovered and exploited to transfer biologically active molecules into cells^1^,^2^,^3^,^4^. However, these techniques have significant drawbacks in their efficiency, cytotoxicity and convenience. In the stem cell research field, it is important that the intracellular delivery system is safe and available for clinical application, as these techniques may help cure many human diseases. For example, protein delivery in stem cells is considered a relatively safe treatment strategy in regenerative medicine because transient gene regulation does not require or induce any genomic alterations.

Since the first report in 1994^5^, cell-penetrating peptides (CPPs) have been considered a promising delivery system, and there are currently several different methods of CPP intracellular delivery. The CPP also called protein transduction domains (PTDs) can deliver many types of cargo, such as oligonucleotides, small molecules, siRNA, nanoparticles, peptides and proteins, into cells^6^,^7^,^8^,^9^,^10^. Generally, CPPs consist of short basic amino acid sequences with a net positive charge (usually lysine and arginine residues). This type of CPPs are categorized as cationic CPPs^11^, which have the benefit of being able to translocate into the intracellular compartment without causing any cell membrane damage, resulting in low cytotoxicity and high uptake efficiency^12^. There have been many reports about alterations of gene expression levels with the use of CPP-mediated exogenous factor delivery^13^. We also reported previously that the CPP-conjugated coactivator-associated arginine methyltransferase 1 (CARM1) protein can be delivered into human bone marrow stromal cells (hBMSCs, also known as bone marrow-derived mesenchymal stem cells) efficiently and change the global gene expression profiles through modulation of histone modifications^14^.

Recent studies in the development and understanding of CPPs have been performed using various approaches. However, the efficiency and intracellular protein uptake of CPP delivery systems have been difficult to measure accurately. Thus, in the present study, we performed a comparison study to analyze the efficiency between two well-known protein delivery systems, CPP-conjugated and streptolysin O (SLO)-mediated systems. Interestingly, it has been reported that treatment with SLO, a bacterial endotoxin produced by *Streptococcus pyogenes*, can form large pores in the plasma membrane of mammalian cells and may provide the possibility of exogenous protein delivery into the cytosol^15^. Therefore, SLO is being used widely as a material for the delivery of exogenous protein in many fields^16^,^17^,^18^. In fact, several reports regarding SLO-mediated cellular extracts and protein delivery were reported recently in the stem cell research field^19^,^20^.

In this study, we synthesized green fluorescent protein (GFP) and estrogen-related receptor β (ESRRB) with or without CPP-conjugation, and both proteins were transported into hBMSCs and human testicular stromal cells (hTSCs) using these two different protein delivery systems. GFP was used to estimate protein transduction efficiency, cytotoxicity, and intracellular protein uptake rate. In addition, we used ESRRB, which is known to interact with pluripotency-related factors such as OCT4, SOX2, and NANOG to analyze the biological activity.

## Results

### Purification of CPP-conjugated and non-conjugated GFP and ESRRB proteins

**Figure 1:**
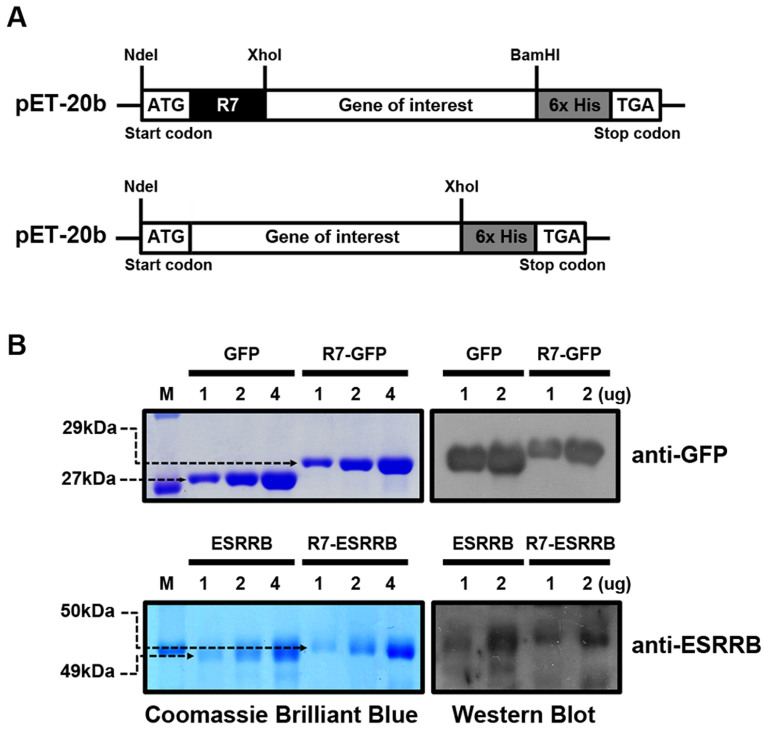
Characterization of recombinant proteins. (A) Schematic diagrams of recombinant proteins with or without the CPP (R7)-conjugated vectors. (B) Identification of recombinant protein (GFP and ESRRB) and R7-conjugated protein (R7-GFP and R7-ESRRB) by Coomassie Brilliant Blue staining and Western blotting using specific antibodies against GFP and ESRRB. Full-length gel and blot images are available in Supplementary Figure 4. M, molecular weight marker.

The ESRRB- and GFP-expressing vectors were constructed by cloning the cDNAs of both factors into the pET-20b vector for purification of the recombinant proteins. Each expression vector construct was transformed into BL21(DE3)pLysiS competent cells, and recombinant proteins were obtained from the soluble fraction. CPP-GFP was confirmed by Coomassie Brilliant Blue staining and detected by a GFP-specific antibody at the molecular weight (MW) of 29 kDa (Fig. 1B). GFP was detected at the MW of 27 kDa because the 7× arginine and 6× histidine sequence was removed. In comparison with the purchased commercial GFP (Atgen), R7-GFP was well enriched. These results demonstrated that the protein purification system was optimized well. CPP-ESRRB and ESRRB were purified using the same procedure as for CPP-GFP. CPP-ESRRB and ESRRB were purified as pure proteins and confirmed by Coomassie Brilliant Blue staining and Western Blot. A specific band was detected at approximately 50 kDa (Fig. 1B). The MW of the ESRRB protein was 1 kDa smaller than that of R7-ESRRB because the 7× arginine sequence was removed.

### Comparison of the protein uptake efficiency using two different protein delivery systems

**Figure 2:**
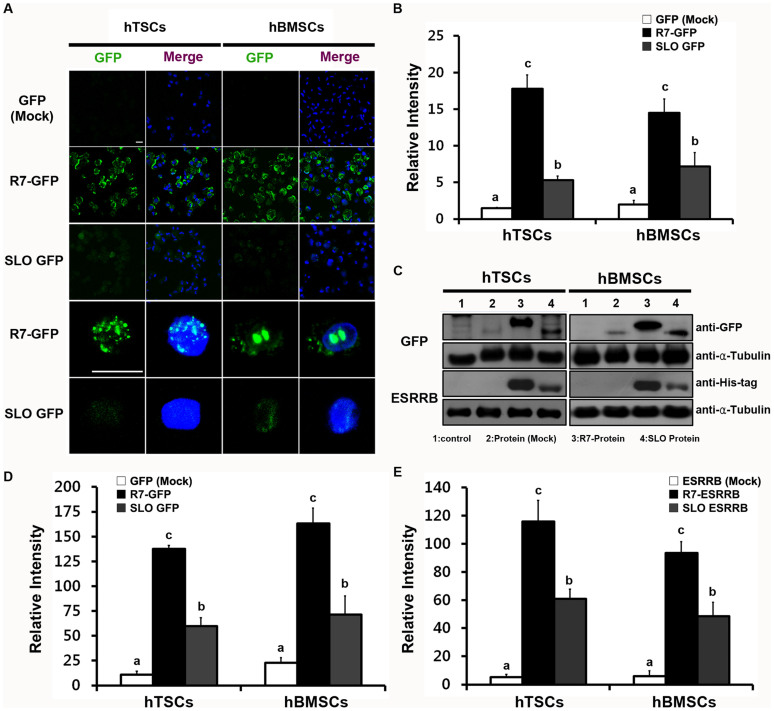
Comparison of the efficiency of two different protein delivery systems (CPP- and SLO-mediated). (A) Transduction of GFP and R7-GFP were detected by confocal microscopy. GFP or R7-GFP were visualized in green. Nuclei were counterstained with DAPI and the images were merged (The top 3 rows show 400× magnification and the bottom 2 rows show 1000× magnification plus 3× zoom). Scale bars represent 20 μm. (B) Relative intensity of GFP. (C) Quantification of delivery of protein (GFP and ESRRB) and CPP-conjugated protein (R7-GFP and R7-ESRRB) were confirmed by Western blot. Lane 1, non-treated control; Lane 2, mock protein control (GFP and ESRRB); Lane 3, CPP-conjugated protein (R7-GFP and R7-ESRRB); Lane 4, SLO-mediated protein (GFP and ESRRB). All samples were normalized to α-Tubulin. Full-length blot images are available in Supplementary Figure 4. Relative intensities are shown for both GFP (D) and ESRRB (E). Data are presented as means ± SEM of three replicates. ^a,b,c^ Different superscripts represent significant differences (*p* < 0.05).

To examine the intracellular protein uptake of the two delivery systems, we performed the following experiments. Suspended hTSCs and hBMSCs were treated with 10 μg of CPP-GFP for CPP-conjugated protein delivery system or with 10 μg of GFP for 1 hr after SLO-mediated pore formation for 50 min for SLO-mediated protein delivery system; the protein uptake efficiency was then compared. In terms of uptaking percentage of cells, we observed a high efficiency of protein delivery both of two different protein delivery systems, nearly 99%. Hence, we performed amount quantification of total intracellular protein uptake by confocal laser microscopy analysis. As shown in Figure 2A, CPP-conjugated GFP was delivered efficiently into hTSCs and hBMSCs. However, the SLO-mediated GFP showed lower delivery efficiency compared with CPP-conjugated GFP. Semi-quantitative analysis of GFP uptake was performed by measuring the intensity of the intracellular GFP signal using ImageJ software (NIH). The intensity of the CPP-GFP signal was over 2-fold that of the SLO-mediated GFP signal in hTSCs and hBMSCs (Fig. 2B).

The intracellular distribution of GFP was examined in high magnification images, and the GFP signal was observed in the nuclei and cytosol (Fig. 2A). Difference in quantity of the two delivery methods was confirmed by Western Blot analysis, and these results were similar to those of the confocal microscopy image data (Fig. 2C–E). To compare the delivery efficiency of a large protein, 50 kDa ESRRB was transduced into hTSCs and hBMSCs. Although ESRRB weighs 2-fold more than GFP, the delivery efficiency was not decreased when compared with that of GFP delivery. Additionally, cellular uptake of the CPP-ESRRB protein was more efficient than that of the SLO-mediated ESRRB protein (Fig. 2A–E).

### *In vitro* cytotoxicity assay

**Figure 3:**
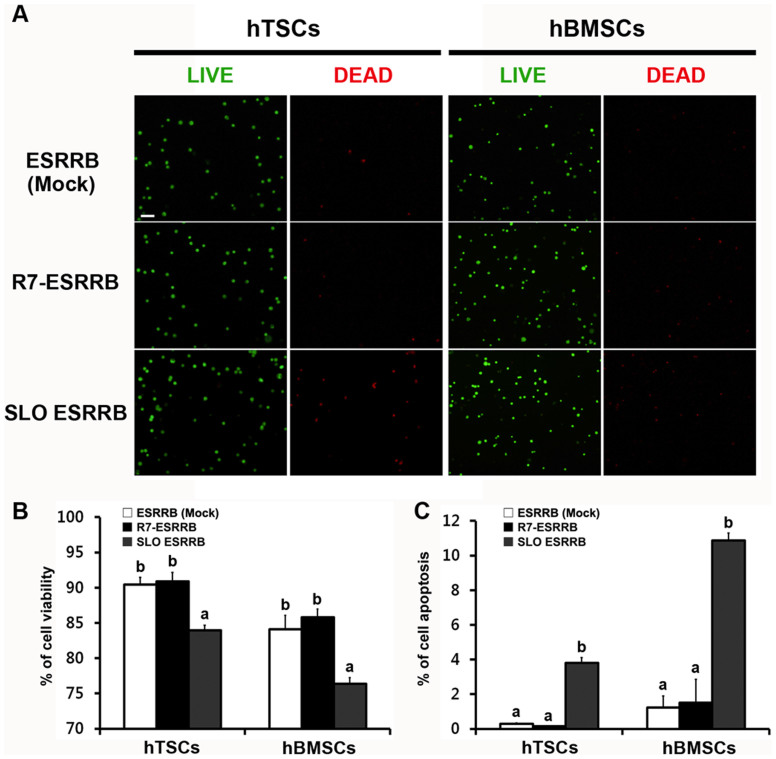
Cytotoxicity assay. (A) Cell viability assay; Live cells (green) and dead cells (red) were detected by fluorescence microscopy. (B) Live cells and dead cells were counted, and the ratio of live to dead cells was calculated. (C) Apoptosis was examined by the TUNEL assay. Scale bars represent 100 μm. Data are presented as means ± SEM of six (B) and three (C) replicates. ^a,b^ Different superscripts represent significant differences (*p* < 0.05).

We evaluated the cytotoxicity of the two protein delivery systems using two different assays. First, we performed a cell viability assay. Live cells were detected with calcein-AM (green signal), and dead cells were detected with ethidium homodimer-1 (red signal) (Fig. 3A). The viability of the CPP-conjugated protein delivery system was 90.0% ± 1.26 in hTSCs and 85.9% ± 1.10 in hBMSCs, compare to the control. However, the viability of the SLO-mediated protein delivery system was 84.0% ± 0.70 in hTSCs and 76.4% ± 0.85 in hBMSCs, indicating that the pore-forming toxin significantly reduces cellular viability (Fig. 3B). Second, we investigated cell apoptosis by the TUNEL assay. The CPP-conjugated protein delivery system induced apoptosis in only a few cells, but the SLO-mediated protein delivery system induced apoptosis in over 4% and 10% of cells (Fig. 3C).

### Comparison of the biological activity of ESRRB delivered cells using two different protein delivery systems

**Figure 4:**
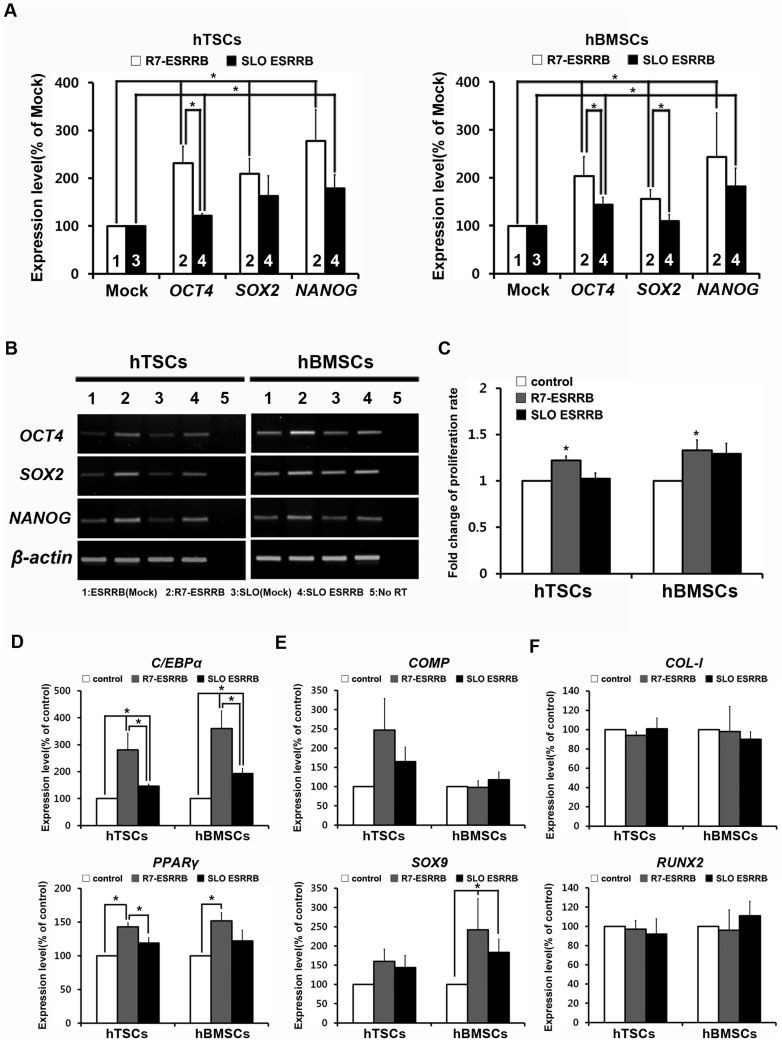
ESRRB induced expression level of pluripotency-related genes, proliferation, and in vitro differentiation efficiency. (A) Quantitative real-time RT-PCR and (B) RT-PCR analysis of pluripotency-related genes. Expression levels of *OCT4*, *SOX2*, and *NANOG* in hTSCs and hBMSCs were analyzed 24 hr after R7-ESRRB or SLO-mediated ESRRB delivery. Each gene was normalized to *β-actin* as a housekeeping control [1: ESRRB(Mock), 2: R7-ESRRB, 3: SLO(Mock), 4: SLO ESRRB]. (C) Altered proliferation rate of ESRRB delivered hTSCs and hBMSCs were calculated as fold change of population doubling number. In vitro differentiation analysis of ESRRB delivered cells was performed by quantitative real-time RT-PCR of (D) adipogenesis-specific markers *C/EBPα* and *PPARγ*, (E) chondrogenesis-specific markers *COMP* and *SOX9*, and (F) osteogenesis-specific markers *COL-I* and *RUNX2*. Data are presented as means ± SEM of four (A), six (B), three (C), and four (D–F) replicates. * *p* < 0.05.

Finally, to compare the biological activity of CPP-ESRRB and SLO-mediated ESRRB delivery, hTSCs and hBMSCs were treated, and the cells were collected 24 hr after delivery. The biological activity of ESRRB was measured as the alteration of the expression levels of pluripotency-related genes using qRT-PCR. The expression levels of *OCT4*, *SOX2*, and *NANOG* showed a significant increase in the CPP-ESRRB delivered cells compared with cells treated by SLO-mediated ESRRB-delivery (Fig. 4A, B). In addition, to measure biological activity of ESRRB in other way, proliferation rate of control, CPP-conjugated and SLO-mediated ESRRB delivered hTSCs and hBMSCs were calculated. The groups of CPP-ESRRB delivered hTSCs and hBMSCs showed a significantly increased proliferation rate (Fig. 4C). Also, differentiation potential was examined to detect biological activity of delivered ESRRB. All three groups of hTSCs and hBMSCs were induced in vitro differentiation into three mesodermal lineage cells; adipogenic, chondrogenic, and osteogenic cells. In adipogenic differentiation, we confirmed elevated expression level of *C/EBPα* and *PPARγ* which are adipogenic-specific markers in both CPP-conjugated and SLO-mediated ESRRB delivered cells compare to control cells. Importantly, CPP-ESRRB delivered hTSCs and hBMSCs showed significant higher expression levels in both markers than SLO-mediate ESRRB delivered cells (Fig. 4D). In case of chondrogenic differentiation, *COMP* and *SOX9* were used as chondrogenesis-specific markers, and CPP-ESRRB delivered hTSCs showed much higher expression level in *COMP* compares to control and SLO-mediated ESRRB delivered cells. Besides, hBMSCs showed significantly increased efficiency in *SOX9* expression level in both CPP- and SLO-mediated ESRRB delivered cells, but significantly higher expression was detected in CPP-ESRRB delivered cells than SLO-mediated ESRRB delivered cells (Fig. 4E). However, we could not find any significant difference in osteogenesis differentiation in all groups (Fig. 4F). Collectively, delivery of the large CPP-ESRRB protein was shown to be efficient and result in measurable levels of biological activity.

## Discussion

CPP has been considered as a safe, convenient, and useful tool for stem cell manipulation by introduction of exogenous protein, and applied in many research, pharmaceutical, and clinical fields. Many researchers have studied using various CPP and there were reports that oligoarginine-conjugated p53 protein can be transduced into cancer cells to inhibit their growth^21^,^22^. However, a few studies were conducted for assessment of CPP-conjugated protein delivery efficiency. In this study, therefore, we performed a comparison assay of two different protein delivery systems using human stromal cells to evaluate the efficiency of those tools, which are CPP- and SLO-mediated protein delivery systems. We have found that the CPP-conjugated protein delivery system has a significantly higher protein uptake efficiency compared to SLO-mediated protein delivery system. Also, CPP-conjugated protein caused few cell damages while SLO showed reduction of cell viability causing apoptotic cell death. The effect of delivered CPP-conjugated protein showed better results than SLO-mediated delivered protein. The bottom line is that CPP-conjugated protein delivery system is regarded as a better than SLO-mediated protein delivery system.

Nevertheless, although oligoarginine is a tremendous protein delivery tool, there is a limitation that individual proteins must be manipulated to link to the CPP domain. SLO is a useful bacterial toxin and a very simple method for exogenous protein delivery, but the SLO-mediated protein delivery system was shown to result in a lower protein uptake rate and higher cytotoxicity. Because the SLO-mediated protein delivery system has the advantage that individual proteins do not need to be manipulated, it has been widely used for delivery of undefined proteins, such as cellular protein extract^19^,^20^. The two different protein delivery systems can be used for their appropriated purposes in many cases. However, protein delivery for clinical applications in stem cell research fields requires defined proteins that are known to regulate the expression of specific genes so that they can control the appropriate cellular properties. Otherwise, unwanted side effects are a considerable risk.

The characteristics of mesenchymal stem cells (MSCs) revolve around multipotency and self-renewal^23^. So, MSCs have great potential as cell therapeutic materials considering their characteristics of self-renewal, proliferation, and differentiation into various cell types^24^,^25^. While MSCs have been spotlighted as an attractive tool for therapeutic application due to their various potentials, there are several challenges for the implementation of MSCs in the clinical setting, such as a limited number of passages *in vitro* and loss of efficient differentiation into multiple lineages^26^,^27^,^28^. To overcome these limitations, many researchers have tried to alter the properties of MSCs by delivery of defined factors using several systems. Among these efforts, several studies have tried that pluripotency-related genes were overexpressed in MSCs to maintain stemness, thus, MSCs quality was improved, thus proliferation and differentiation potential were enhanced^29^,^30^,^31^. These results demonstrated that the expression of pluripotency-related genes can maintain the stemness of MSCs and this type of manipulation could increase efficiency of clinical application required a good quality of MSCs.

In the present study, ESRRB as a defined protein was used to regulate the expression levels of pluripotency-related genes in human mesenchymal stromal cells. ESRRB is an orphan nuclear receptor and is an important factor involved in the maintenance of self-renewal and pluripotency. It mediates the reprogramming of somatic cells to pluripotent stem cells in conjunction with OCT4 and SOX2^32^. Here, we have shown that ESRRB protein delivery into hTSCs and hBMSCs increased the *OCT4*, *SOX2* and *NANOG* expression levels. CPP-conjugated ESRRB delivery presented a more efficient uptake and a more significant increase in the expression levels of *OCT4*, *SOX2* and *NANOG* than SLO-mediated ESRRB delivery. Furthermore, cytotoxicity was shown to be low in the CPP system. CPP-ESRRB delivery may be a good tool for maintenance of the hMSC populations and can thus make them more available for clinical applications. CPP-conjugated proteins can be successfully delivered in adherent cells^14^; therefore, CPP-ESRRB can be used easily as a medium supplement. Continuous CPP-ESRRB intracellular delivery may elevate the proliferation and differentiation potential of human stromal cells. However, in the present study, a single treatment of CPP-ESRRB was performed in suspended human stromal cells because the purpose of the study was to evaluate and compare the CPP- and SLO-mediated delivery systems as potential tools in hMSCs manipulation.

Although oligoarginine is a good tool for protein delivery, it is not as effective as viral gene transfer. However, there was a recent report that protein uptake efficiency was increased using oligoarginine with pyrenebutyrate^33^. Therefore, a safe, convenient, and selective defined protein delivery system must be developed for further clinical application in stem cell research. Our results suggest that CPP-conjugated protein delivery is an excellent tool for biologically active, defined protein delivery and may have important clinical applications for the use of stem cells in regenerative medicine.

## Methods

### Construction of a protein expression vector and purification of proteins

To generate pure protein for intracellular delivery, a protein expression vector was constructed as previously described^14^. Briefly, we modified the pET-20b vector with 7 arginines (R7) at the N-terminal as the CPP sequence and 6× His-tag at the C-terminal. We removed the CPP sequence for the proteins used for SLO-mediated delivery. The gene of interest could easily be inserted into pET-20b using *Nde*I, *Xho*I, and *Bam*HI restriction sites, which facilitate the manipulation of various open reading frames (ORFs; Fig. 1A). The constructed vectors were transformed into BL21(DE3) pLysS competent cells (Stratagene Inc., La Jolla, CA) and cells were cultured in LB medium and induced with 1 mM isopropyl-1-thio-β-D-galactopyranoside (IPTG, Sigma-Aldrich, St. Louis, MO). Cells were lysed with NP-10 [50 mM NaH2PO4 (Sigma Aldrich), 300 mM NaCl (Sigma Aldrich), 10 mM imidazole (Sigma Aldrich)] solution and benzonase (2 units/ml: QIAGEN, Inc., Basel, Switzerland). Proteins in the soluble supernatant were purified by His-Tagged Recombinant Protein Purification - His60 Ni Resin (ClonTech laboratories, CA, USA) according to the manufacturer's instructions. GFP recombinant protein without the R7 domain and 6× His-tag was purchased from Atgen (atgp0302; Seoul, South Korea). The identity of the each purified protein was confirmed by Coomassie Brilliant Blue R250 staining and Western blotting with specific primary antibodies: anti-GFP (AB3080; Millipore, Billerica, MA) and anti-ESRRB (sc-47662; Santa Cruz Biotechnology, Santa Cruz, CA).

### Culture of hTSCs and hBMSCs

We used the hTSCs and hBMSCs. The hTSCs were obtained from excised human testes and were isolated the CD34^+^/73^+^ cells and cultured as previously described^34^. hTSCs have a fibroblastic morphology, similar to hBMSCs (Fig. S1). The hTSCs were positive for CD34, CD73, class I major histocompatibility (MHC) antigens (HLA ABC), CD29, CD44, CD90, CD105, and CD166 and were negative for CD14, CD31, CD45, HLA DR, TRA-1-60, SSEA3, TRA-1-81, c-Kit, CD133, and CD140 (Fig. S2B). This study was approved by the Institutional Review Board of the CHA Gangnam Medical Center, Seoul, South Korea. The hBMSCs were purchased from Lonza (PT2501) and positive for CD105, CD90, CD73, CD166 (data not shown), CD29 (data not shown) and CD44 (data not shown) and were negative for CD14 (data not shown), c-Kit, CD34, CD45, HLA-DR, and SSEA4, as determined by flow cytometry (Fig. S2B; Lonza, Walkersville, MD). The hBMSCs were expanded and cultured in DMEM/F12 (Gibco-BRL) supplemented with 10% FBS (Gibco-BRL) and 100 U/ml penicillin G, and 100 μg/ml streptomycin (Gibco-BRL). Cells were maintained in T75 flasks (Nunc, Roskilde, Denmark) at 37°C in a 5% humidified CO_2_ incubator and subcultured every 4–5 days. In this study, we used the cultured cells at passage 4 ~ 6.

### Direct delivery of exogenous protein into hTSCs and hBMSCs using CPP-conjugated and SLO-mediated systems

GFP and ESRRB proteins were delivered into suspended hTSCs and hBMSCs. Cells released with 0.05% trypsin-EDTA (Hyclone) were collected and washed with Hank's balanced salt solution (HBSS) without Ca^2+^ and Mg^2+^ (Gibco-BRL) and total 7.5 × 10^5^ cells were collected for each group. In the case of SLO-mediated delivery, cells were incubated in 1 ml HBSS with 230 ng SLO, for 50 min at 37°C, and tubes were inverted to mix thoroughly. Then, tubes were incubated on ice for 5 min. After centrifugation, perforated cells were collected, and resuspended in 200 μl HBSS. The protein of 10 μg of GFP or ESRRB were treated for 1 hr at 37°C. After washing with HBSS, resealing of pores was induced by the addition of 2 mM CaCl_2_ in 1 ml growth medium for 2 hr.

In the CPP-conjugated protein delivery system, cells were suspended in 200 μl HBSS with 10 μg of CPP-GFP or CPP-ESRRB and incubated for 1 hr at 37°C. After that, cells were washed with an acidic buffer to remove the CPP-protein that was absorbed on cell surface. Cells were washed twice for 30 sec with cold 0.2 M glycine buffer containing 500 μl of 0.15 M NaCl (pH 3.0), followed by DPBS wash^35^. GFP protein was used for visualization of protein delivery, and we evaluated the protein transduction efficiency, intracellular protein uptake, and localization. Therefore, GFP or CPP-GFP protein-treated cells were sampled immediately and assessed. The purpose of using ESRRB was to provide a functional assay to estimate the biological activity of proteins. Thus, ESRRB or CPP-ESRRB protein-delivered cells were seeded onto 6-well dishes, and after 24 hr in culture, the cells were collected for further experiments.

### Confocal laser microscopic analysis

After protein delivery using CPP-GFP or SLO-mediated GFP, each sample was split at a count of 1 × 10^5^ cells and fixed in 4% paraformaldehyde (PFA). The cells were attached to glass slides by cytospin at 1000 rpm for 5 min. All samples were counterstained with 4′,6-diamidino-2-phenylindole (DAPI; Sigma Aldrich). All images were captured with a Carl Zeiss LSM 510 META confocal laser-scanning microscope (Carl Zeiss, Jena, Germany). The intensity of GFP was measured using ImageJ software (NIH; http://rsb.info.nih.gov/ij/).

### Quantification of delivered protein by Western Blot analysis

Western blot analyses were performed for quantification of the delivered proteins in the cells. Each sample contained 1 × 10^4^ cells, protein extracts were obtained using the PRO-PREP (iNtRON Biotechnology, Seoul, South Korea) according to the manufacturer's instructions. Each sample was quantified using the Quant-iT Protein assay kit (Invitrogen), and protein extracts were separated by 10 ~ 12% SDS-PAGE and transferred onto polyvinylidene difluoride membranes (Bio-Rad Laboratories, Hercules, CA). The membranes were incubated with specific primary antibodies. Immunoreactivity was detected using the Western Blotting Luminol Reagent (Santa Cruz Biotechnology), and membranes were developed on Amersham Hyperfilm ECL X-ray film (Amersham Pharmacia Biotech, Buckinghamshire, UK). Each group was normalized to α-tubulin. The following antibodies were used for Western blot analyses: anti-α-tubulin (Sigma Aldrich, T9026), anti-ESRRB (Santa Cruz Biotechnology, sc-47662), anti-His-tag (Sigma-Aldrich, H1029), and anti-GFP (Millipore, AB3080). The intensity of bands was measured using ImageJ software (NIH) for semi-quantitation.

### Cell viability and apoptosis assay

ESRRB-delivered hTSCs and hBMSCs were stained with the LIVE/DEAD® Viability/Cytotoxicity Kit for mammalian cells (Molecular Probes™, Eugene, OR). Intracellular esterase activity and plasma membrane integrity were detected with two color fluorescence, calcein AM and ethidium homodimer (EthD-1). Calcein is well retained within living cells and expresses green fluorescence; EthD-1 enters cells with damaged membranes and produces red fluorescence in dead cells. Live cells (green) and dead cells (red) were counted, and the ratio was calculated. Apoptosis assay of CPP-conjugated and SLO-mediated protein groups was conducted by deoxynucleotidyl transferase dUTP nick-end labeling (TUNEL) assay. The In Situ Cell Death Detection Kit, TMR red (Roche, Mannheim, Germany) was used to label DNA strand breaks with TMR red. Cells were fixed with 4% PFA and attached to glass slides by Cytospin. All images were photographed using an optical microscope (Eclipse TE-2000 U, Nikon, Tokyo, Japan).

### RNA extraction, RT-PCR, and Real-time RT-PCR

To quantify expression levels of pluripotency-related genes (*OCT4*, *SOX2*, *NANOG*) after ESRRB delivery, real-time RT-PCR was performed. Total RNA was extracted from cells using TRIzol reagent (Invitrogen Carlsbad). First strand cDNA was synthesized with 2 μg of each RNA using a PrimeScript 1^st^ strand cDNA synthesis kit (TakaraBio, Shiga, Japan) according to the manufacturer's instructions. The quantitative real-time RT-PCR was performed using iQTm SYBR Green supermix (Bio-Rad Laboratories) on a Bio-Rad iQ5 real-time PCR machine. The primer sequences for the genes are listed in supplementary table 1. Each gene was normalized to *β-actin* as a housekeeping control. The results were analyzed using the delta-delta Ct method with the use of housekeeping genes^36^.

### Proliferation assay

ESRRB was delivered into hTSCs and hBMSCs using R7-ESRRB or the SLO-mediated protocol, and then each cell line was seeded in a T75 flask at count of 2.2 × 10^5^ cells. After 5 days, each group of cells was collected and the number of cells in each group was counted manually. Then, those cells were used to perform protein delivery and seeded onto T75 flask again as described above. The population doubling number of each subculture was calculated at every subculture with the formula 2^X^ = N_H_/N_I_. N_I_ is the seeded cell number, N_H_ is the harvested cell number, and X is population doubling.

### In vitro differentiation of hTSCs and hBMSCs delivered ESRRB protein

To examine biological activity of delivered protein, we performed the induction to differentiate into adipogenic, chondrogenic, and osteogenic lineages in vitro. Right after delivery of ESRRB, each group of cells was seeded on 6-well plates at a count of 2 × 10^5^ cells per well and grown. At 80% of confluence, growth medium were replaced with either StemPro Adipogenesis Differentiation Kit (Gibco-BRL), StemPro Chondrogenesis Differentiation Kit (Gibco-BRL), or StemPro Osteogenesis Differentiation Kit (Gibco-BRL), and differentiation medium was changed every 3–4 days. Cells were collected after 2–3 weeks of differentiation and total cellular RNA was extracted from each group as described above. Efficiency of differentiation was defined by real-time PCR using lineage specific marker gene, *C/EBPα* and *PPARγ* in adipogenic differentiation, *COMP* and *SOX9* in chondrogenic differentiation, *COL-I* and *RUNX2* in osteogenic differentiation group.

### Statistical analysis

All experiments were performed in at least three independent experiments, and the results are expressed as the mean ± standard error. Statistical analyses were performed using the one-way ANOVA test and followed by Student's *t*-test if necessary. A *p* value of *p* < 0.05 was considered statistically significant.

## Author Contributions

J.J. and D.L. conceived and designed the experiments. J.J., S.H. and W.C. performed experiments and analyzed the results. J.J., S.H. and D.L. discussed the results and wrote the manuscript. D.L. advised the experiments and revised manuscript.

## Supplementary Material

Supplementary Information Supplementary Information
