# Supplementary material for: Cell-penetrating peptide (CPP)-conjugated proteins is an efficient tool for manipulation of human mesenchymal stromal cells
Source: Sci Rep. 2014 Mar 14;4:4378. doi: 10.1038/srep04378 (PMC3953728; doi:10.1038/srep04378)
Supplement: Supplementary Information [file srep04378-s1.pdf]

Title: Cell-penetrating peptide (CPP)-conjugated proteins is an efficient tool for manipulation of human mesenchymal stromal cells

Names of authors:

Junghyun Jo [a,1], Soomin Hong [a,1], Won Yun Choi [b,c], and Dong Ryul Lee [a,b,d,*]

Affiliations of authors:

[a] Department of Biomedical Science, College of Life Science, CHA University, Seoul, Korea

[b] Fertility Center, CHA Gangnam Medical Center, College of Medicine, CHA University, Seoul, Korea

[c] Department of Biotechnology, Seoul Women's University, Seoul, Korea

[d] CHA Stem Cell Institute, CHA University, Seoul, Korea

[1] These authors contributed equally to this study.

[*] *To whom correspondence may be addressed:* **Dong Ryul Lee, Ph.D.,** Department of Biomedical Science, College of Life Science, CHA University, 606-13 Yeoksam-dong, Gangnam-gu, Seoul 135-081 Korea. Tel: 82-2-3468-3421; E-mail: drleedr@cha.ac.kr

SUPPLEMENTARY MATERIALS AND METHODS

Cumulative population doubling number assay

The population doubling (PD) number was calculated at every subculture with the formula $2^X = N_H/N_I$. N_I is the seeded cell number, N_H is the harvested cell number at 80% of confluence, and X is population doubling. Each PD number was added to the previous PD number to yield cumulative population doubling number.

Flow cytometric analysis

To identity of hBMSCs and hTSCs characteristics, flow cytometric analysis was performed. hBMSCs and hTSCs were dissociated to single cells by treatment of 0.05% Trypsin-EDTA (Hyclone) for 3 min. Each group of cells was fixed with 4% paraformaldehyde (PFA) for 10 min and treated with appropriate antibodies. Fixed hBMSCs were incubated with primary antibodies, CD34, CD45, CD73, CD90, CD105, HLA-DR, SSEA4 (BD), and c-Kit (Santa Cruz Biotechnology) for 20 min at 4℃ in the dark. hTSCs were treated with CD14, CD29, CD31, CD34, CD44, CD45, CD73, CD90, CD105, CD140 (BD), CD133 (eBioscience,Inc.), CD166, HLA-ABC, HLA-DR, TRA-1-60, SSEA3, TRA-1-81 (BD), and c-Kit (Santa Cruz Biotechnology) for 20 min at 4℃ in the dark. Then, cells were washed three times with Ca^{2+}, Mg^{2+}-free PBS (Hyclone). Cells then washed twice with PBS (Hyclone) and suspended in fluorescence-activated cell sorting (FACS) buffer (1x PBS with 2% FBS), and filtered through 5 ml polystyrene round-bottom tube with a cell-strainer cap (12x75 mm style, BD Bioscience). Flow cytometric analysis was performed using a FACS Calibur Flow Cytometer (FACS Vantage SE System; BD) and Cell Quest Pro software (BD Bioscience).

In vitro differentiation for adipogenic, chondrogenic, and osteogenic cells

To analyze hBMSCs and hTSCs differentiation potential into mesodermal-lineage cells, in vitro differentiation was performed. hBMSCs and hTSCs were plated onto 6-well culture dishes and maintained with growth medium until 80% of confluence. Then, cells were induced to differentiate by replacement of medium with adipogenic, chondrogenic, and osteogenic differentiation medium (Invitrogen) and grown in these medium for 21 days. Differentiated adipogenic cells were fixed with 10% formalin for 30 min at room temperature (RT) and then washed with distilled water. Adipogenic differentiation was visualized using Oil Red O (Sigma-Aldrich) staining and cells were stained with an oil red O working solution for 15 min at RT. Differentiated chondrocytes were fixed with 4% PFA for 30 min at RT. To visualize the chondrocytes, we used 1% Alcian Blue (Sigma-Aldrich) in 0.1 N HCl. Differentiated osteogenic cells were fixed with 4% PFA for 30 min at RT, and stained with 2% Alizarin Red (Sigma-Aldrich). Also, all three types of differentiated cells were harvested and total RNA was extracted to perform real-time PCR. The expression of adipogenesis-specific markers,

C/EBPα and *PPARγ*, were analyzed, and chondrogenesis-specific markers such as *COMP* and *SOX9* were examined. To measured osteogenesis-specific markers expression, we used *COL-I* and *RUNX2*.

SUPPLEMENTARY FIGURE

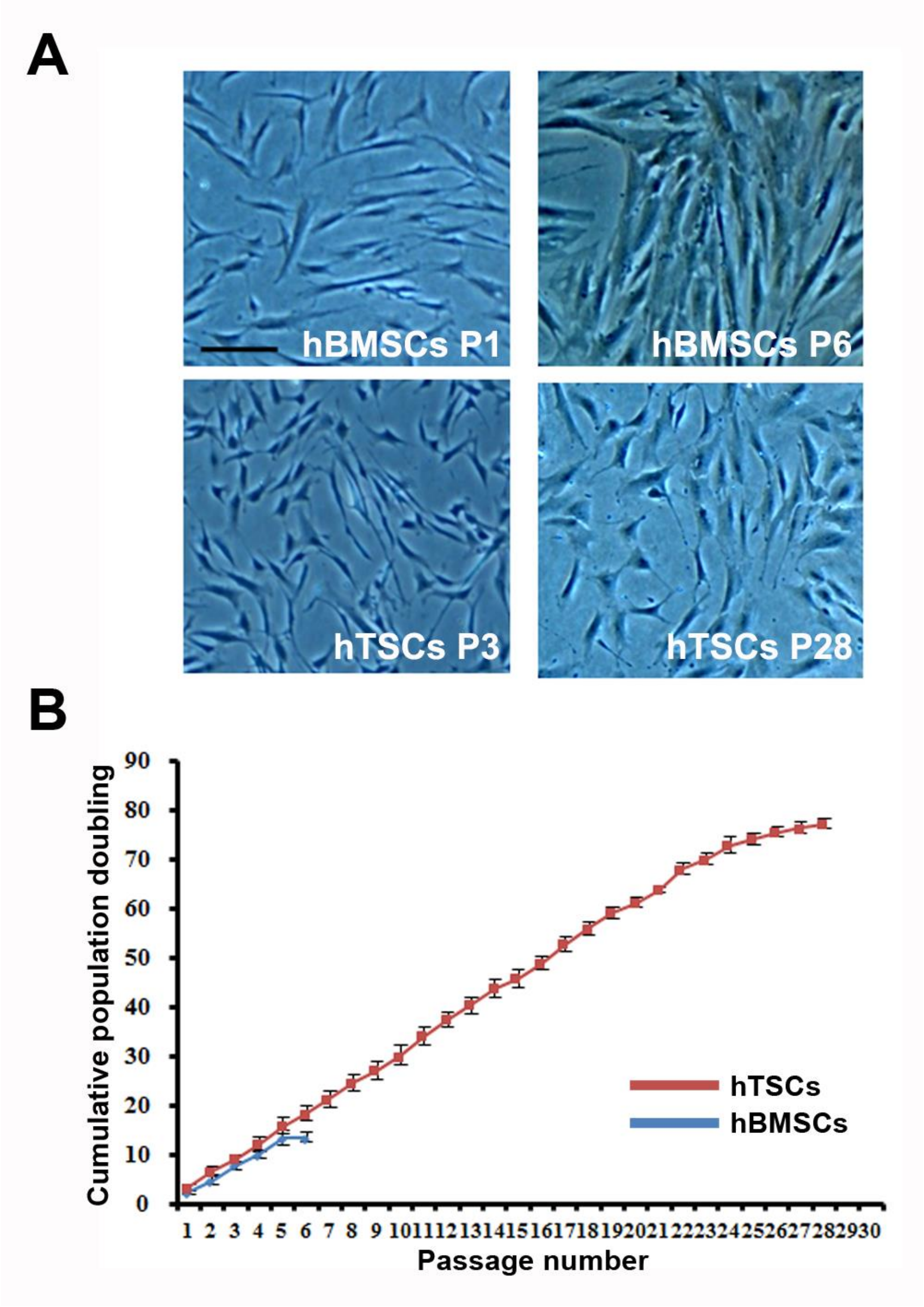

Supplementary Figure 1. Morphological and proliferative characteristics of hBMSCs and hTSCs. (A) Phase-contrast images of hBMSCs and hTSCS. hBMSCs at P1 showed MSC-like morphology but at P6 showed senescent-like morphology. hTSCs were at P3 and P28 with the same morphology. (B) Cumulative population doubling numbers of hBMSCs and hTSCs from P1 to P30. Scale bars indicates 50 μm.

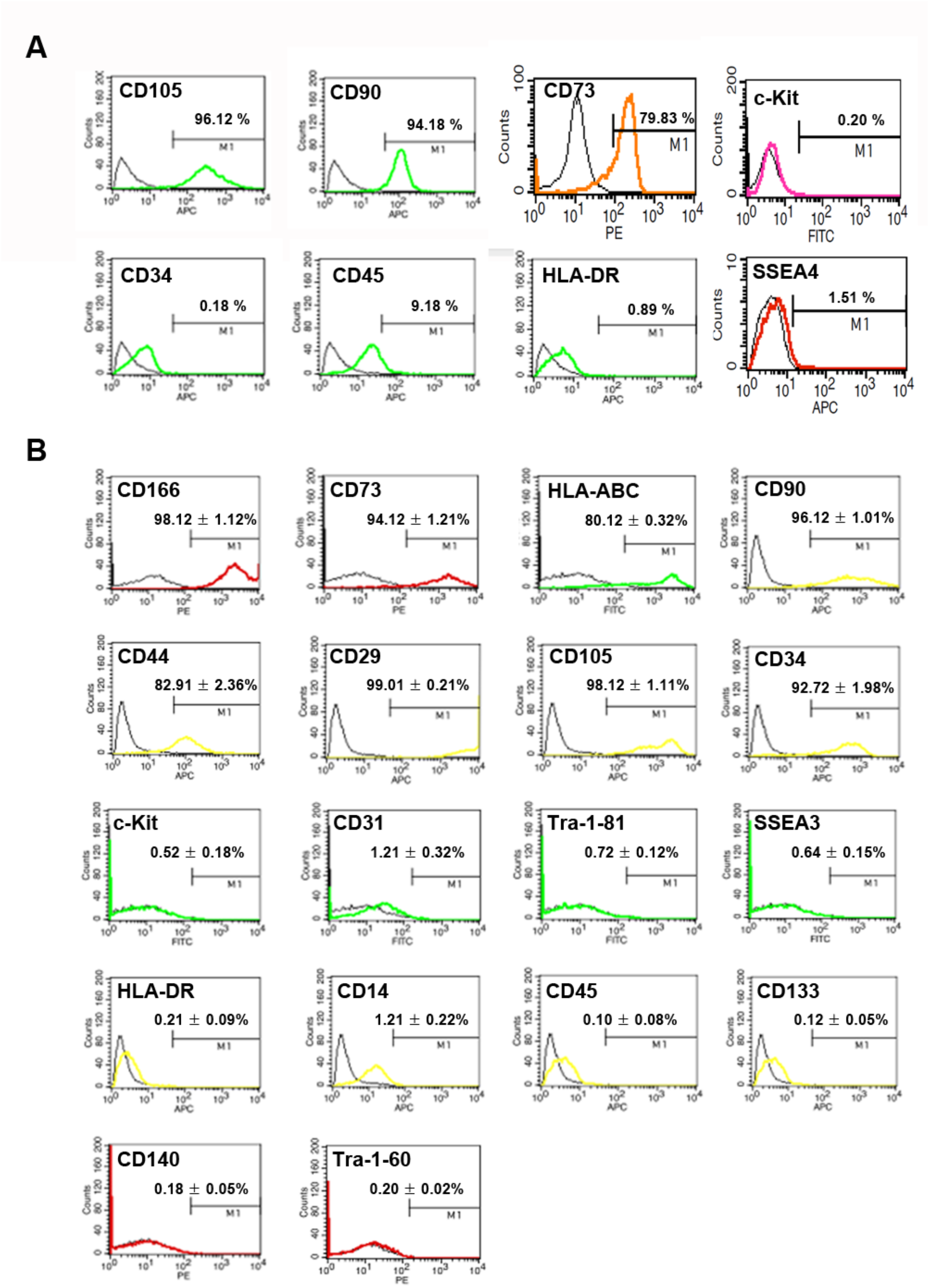

Supplementary Figure 2. Characterization of hBMSCs and hTSCs by flow cytometric analysis. (A) hBMSCs showed positive for CD105, CD90, CD73, and c-Kit, but negative for CD34, CD45, HLA-DR, and SSEA4. (B) hTSCs expressed CD166, CD73, HLA-ABC, CD90, CD44, CD29, CD105, and CD34. But c-Kit, CD21, Tra-1-81, SSEA3, HLA-DR, CD14, CD45, CD133, CD140, and Tra-1-60 was not detected.

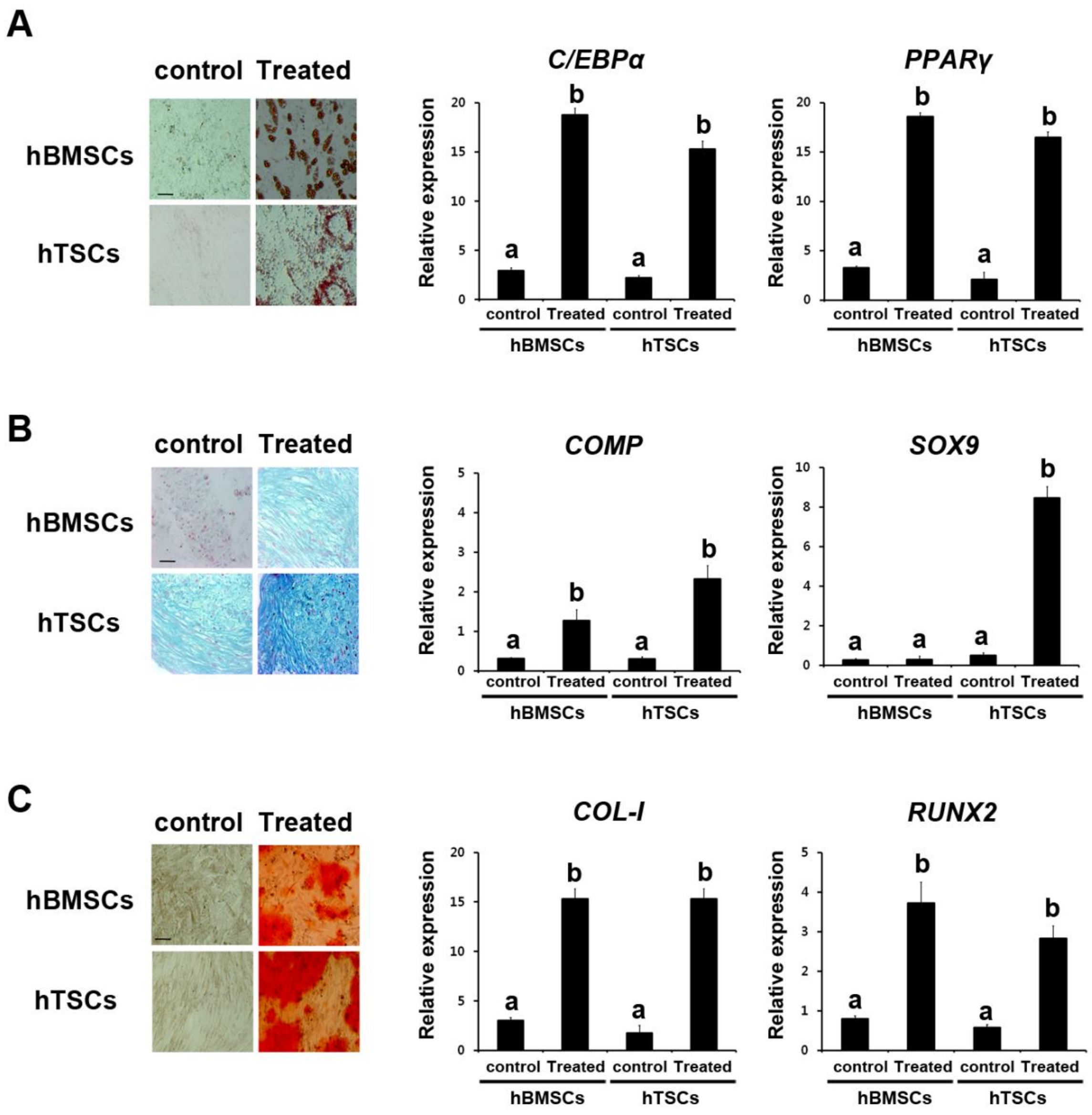

Supplementary Figure 3. Characterization of mesodermal differentiation potential in hBMSCs and hTSCs. Control cells were stated as control, and differentiation medium treated cells were clared as Treated. (A) Lipid droplets were stained with Oil Red O in hBMSCs and hTSCs. Adipogenesis-specific markers *C/EBPα* and *PPARγ* were examined by real-time PCR. (B) Alcian blue staining was performed with both cell lines for detection of sulfated proteoglycans after chondrogenic differentiation. Chondrogenesis-specific markers, *COMP* and *SOX9*, expression level were measured by real-time PCR. (C) Alizarin Red was used to stain calcium deposits in case of osteogenic differentiation, and osteogenesis-specific markers such as *COL-I* and *RUNX2* were used for real-time PCR. Scale bars indicate 100 µm.

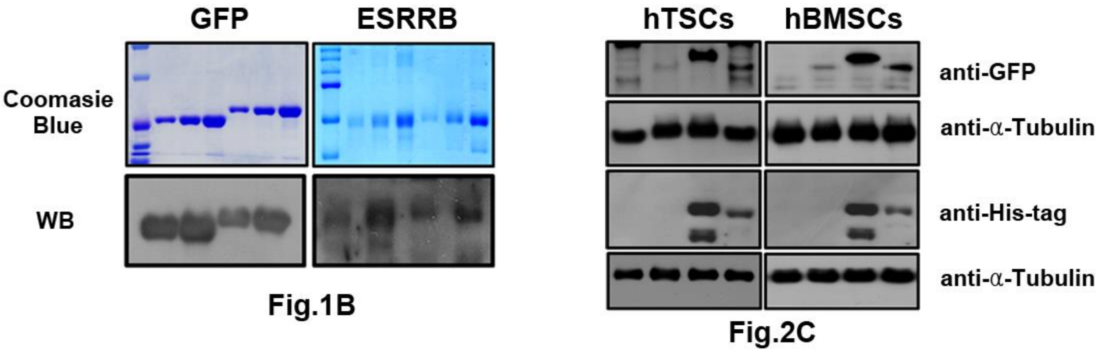

Supplementary Figure 3. Full-length gels and blots images. Full-length gel and blot images for Fig.1B and Fig.2C

Supplementary Table 1. Primer sequences for plasmid construction and PCR.

Gene	Purpose	Sequences		Annealing Temp. (℃)	Product size (bp)
ESRRB	Cloning	F:	5'-CTCGAGATGTCGTCCGAAGACAGGCAC-3'	58	1,311
	with CPP	R:	5'-GGATCCCACCTTGGCCTCCAGCATCTC-3'		
ESRRB	Cloning	F:	5'-CATATGTCGTCCGAAGACAGGCACCTG-3'	58	1,308
	without CPP	R:	5'-CTCGAGCACCTTGGCCTCCAGCATCTC-3'		
GFP	Cloning	F:	5'-CTCGAGATGGTGAGCAAGGGCGAGGAG-3'	58	729
	with CPP	R:	5'-GGATCCCTTGTACAGCTCGTCCATGCC-3'		
OCT4	RT-PCR	F:	5'-AGCGAACCAGTATCGAGAAC-3'	60	142
	qRT-PCR	R:	5'-TTACAGAACCACACTCGGAC-3'		
SOX2	RT-PCR	F:	5'-AGCTACAGCATGATGCAGGA-3'	60	126
	qRT-PCR	R:	5'-GGTCATGGAGTTGTACTGCA-3'		
NANOG	RT-PCR	F:	5'-TGAACCTCAGCTACAAACAG-3'	60	154
	qRT-PCR	R:	5'-TGGTGGTAGGAAGAGTAAAG-3'		
C/EBPα	*RT-PCR*	F :	5'-GCAAACTCACCGCTCCAATG-3'	57	247
	qRT-PCR	R :	5'-TTAGGTTCCAAGCCCCAAGTC-3'		
PPARγ	*RT-PCR*	F :	5'-TGTCTCATAATGCCATCAGGTTTG-3'	57	224
	qRT-PCR	R :	5'-GATAACGAATGGTGATTTGTCTGTT-3'		
COMP	*RT-PCR*	F :	5'-AACGCTGAAGTCACGCTCAC-3'	60	244
	qRT-PCR	R :	5'-GGTAGCCAAAGATGAAGCCC-3'		
SOX9	*RT-PCR*	F :	5'-TTCATGAAGATGACCGACGA-3'	60	326
	qRT-PCR	R :	5'-CACACCATGAAGGCGTTCAT-3'		
COL-I	*RT-PCR*	F :	5'-AGAACATCACCTACCACTGC-3'	57	250
	qRT-PCR	R :	5'-ATGTCCAAAGGTGCAATATC-3'		
RUNX2	*RT-PCR*	F :	5'-CCCCACGACAACCGCACCAT-3'	61	289
	qRT-PCR	R :	5'-CACTCCGGCCCACAAATCTC-3'		
β-actin	RT-PCR	F:	5'-TGAAGTGTGACGTGGACATC-3'	60	152
	qRT-PCR	R:	5'-GGAGGAGCAATGATCTTGAT-3'		
